# Effect of Socio-Economic Status on Perioperative Outcomes After Robotic-Assisted Pulmonary Lobectomy

**DOI:** 10.7759/cureus.26201

**Published:** 2022-06-22

**Authors:** Anastasia Jermihov, Liwei Chen, Maria F Echavarria, Emily P Ng, Frank O Velez, Carla C Moodie, Joseph R Garrett, Jacques P Fontaine, Eric M Toloza

**Affiliations:** 1 School of Medicine, University of South Florida Health Morsani College of Medicine, Tampa, USA; 2 Surgery, University of South Florida Health Morsani College of Medicine, Tampa, USA; 3 Thoracic Oncology, Moffitt Cancer Center, Tampa, USA; 4 Surgery and Oncologic Sciences, University of South Florida Health Morsani College of Medicine, Tampa, USA

**Keywords:** socio-economic status, perioperative outcomes, minimally invasive surgery, robotic surgery, pulmonary lobectomy

## Abstract

Background: Lower socioeconomic status (SES) has been correlated with poor survival rates and surgical outcomes following lung cancer resection. This study sought to determine whether this disparity exists perioperatively in lung cancer patients following robotic-assisted video-thoracoscopic pulmonary lobectomy.

Methods: We retrospectively reviewed 447 consecutive patients who underwent robotic-assisted pulmonary lobectomy by one surgeon for known or suspected lung cancer. Ten patients were excluded due to incomplete data. We used median income by residential ZIP code as a surrogate for SES status and grouped patients based on whether ZIP-based median income was less than (Group 1) or greater than (Group 2) 300% of the federal poverty income level. The effects of SES status groups on incidence of postoperative complications, chest tube duration, hospital length of stay (LOS), and in-hospital mortality were evaluated by the logistic regression model and Inverse Gaussian regression model, respectively.

Results: Without adjustment, Group 1 tended to have a higher rate of postoperative complications, with 54% of patients experiencing complications compared to 34% of patients in Group 2 (p=0.007). Median chest tube duration and hospital LOS were also significantly longer in Group 1 than in Group 2 (p=0.034). In multivariable logistical regression analysis, while controlling for covariates and considering effect modifications, lower SES was significantly and positively associated with postoperative complications (odds ratio (OR)=1.98, p=0.039). Preoperative chronic obstructive pulmonary disease (COPD) was also a positive and significant predictor of postoperative complications (OR=1.89, p=0.017), chest tube duration (p=0.020), and LOS (p=0.010).

Conclusions: Lower median income is associated with a greater number of postoperative complications following pulmonary resection for lung cancer when controlling for covariates.

## Introduction

Lung cancer is the most common cause of cancer death in the United States (USA/US) and worldwide, claiming the lives of an estimated 1.6 million individuals each year [1,2]. Approximately 85% of lung cancer patients will be diagnosed with the histological subgroup of non-small cell lung cancer (NSCLC) [2], of which one-fifth will have early-stage (American Joint Committee on Cancer (AJCC) stage-I and -II) disease [1] The recommended treatment for early-stage lung cancer is surgical resection, which means that improving perioperative outcomes is an integral part of disease management.

Lower socioeconomic status (SES) has been shown to negatively influence outcomes in lung cancer patients undergoing lobectomy and other forms of surgical resection and has been associated with shorter median survival time and increased rates of in-hospital mortality and risk-adjusted mortality [3-5]. Patients with low SES demographics, such as low income, minority ethnicity, public insurance coverage, and lower levels of education, have been demonstrated to be less likely to undergo minimally invasive surgery (MIS) [6-9]. One study investigating the influence of insurance coverage on perioperative and long-term outcomes following robotic-assisted video thoracoscopic (RAVT) surgery, a form of MIS for lung cancer, found that patients with public insurance had less favorable outcomes compared to patients with private or combination insurance [10]. Since insurance coverage represents a component of SES, further research is needed to further characterize the influence of SES on outcomes after MIS for lung cancer.

Our study aims to add to the literature on the influence of SES on surgical outcomes, specifically, on MIS outcomes. The question is whether patients, who come from low SES communities and who do undergo MIS, continue to experience the adverse surgical consequences that have been associated with low SES. Area-based indicators, such as ZIP codes, have been strongly correlated with self-reported educational attainment and found to consistently detect associations between low SES and poor clinical outcomes [11]. Median income can be estimated using ZIP-code census data, and income has been identified as a significant and independent predictor of in-hospital mortality and overall survival (OS) following lung cancer resection [4,5]. Interestingly, a study investigating the degree to which individual and neighborhood SES of Black adults affect outcomes in chronic obstructive pulmonary disease (COPD) found that neighborhood-level SES, such as median household income, explained a greater percentage of racial disparities in respiratory outcomes than individual-level SES [12].

RAVT surgery is a newer modality of minimally invasive technique for lung cancer surgery comparable to conventional video-assisted thoracoscopic (VATS) surgery and is associated with reduced intraoperative blood loss and improved surgical precision [13,14]. This study aims to investigate the influence of SES on perioperative outcomes following MIS for lung cancer in the form of RAVT pulmonary lobectomy. We present the protocol in accordance with the Strengthening the Reporting of Observational Studies in Epidemiology (STROBE) reporting checklist.

This study was presented in part at the 15th Annual Academic Surgical Congress in Orlando, Florida (FL), USA, in February 2020.

## Materials and methods

We retrospectively reviewed 447 consecutive patients who underwent RAVT pulmonary lobectomy by one surgeon from September 2010 through August 2018 at a single institution, Moffitt Cancer Center, Tampa, FL, USA. This database protocol was approved by the Scientific Review Committee of Moffitt Cancer Center, Tampa, FL, USA (MCC #16728, #18761, and #19304) and by the Institutional Review Board (IRB) of the University of South Florida, Tampa, FL, USA (USF IRB #Pro00022263) and Chesapeake IRB (now Advarra), Columbia, Maryland (MD), USA (#Pro00017745 and #00000790), which waived informed consent for this retrospective study, which is considered as a review of existing data. Additionally, the patients reviewed for this study all gave informed consent for fiberoptic bronchoscopy, RAVT wedge resection and/or RAVT (completion) lobectomy, mediastinal lymph node dissection (MLND), and possible thoracotomy, the details of which have been previously described [10]. Some patients also gave informed consent for any anticipated *en bloc* chest wall and/or vertebral resection, with possible chest wall and/or vertebral reconstruction. Through the institutional surgical informed consent, patients gave permission to use surgery-related and tissue-related data for education and research purposes.

We used median income by residential ZIP code as a surrogate for SES, because individual income was not available. Median income census data by ZIP code was found using the American FactFinder website powered by the US Census Bureau [15]. Of the 447 consecutive patients over the 94-month period, 10 patients were excluded due to a lack of median income census data for their residential ZIP codes. Patients were grouped based on whether ZIP-based median income was less than (Group 1) or greater than (Group 2) 300% of the federal poverty income level (i.e., “below-3x-poverty” vs. “above-3x-poverty”, respectively). We used the federal poverty level (FPL) for a one-person household in the year 2018, which was $12,140 according to the US Department of Health and Human Services [16]. This value of 300% of the FPL was used because there were very few patients with ZIP-based median incomes that fell below the FPL, so the cut-off was raised in order to increase the number of patients in the low-income study group enough to allow statistical comparison to the higher-income control group. A one-person household income was used based on the assumption that most patients in our study are past retirement age and could be studied as having the income of a one-person household.

In addition to the independent variable of SES by median income, other variables were analyzed, including age, gender, body surface area (BSA), body mass index (BMI), and forced expiratory volume in one second as a percentage of predicted (FEV1%) at surgery. Diffusion capacity of the lung for carbon monoxide (DLCO) was not included in our study, because not enough patients had this value recorded in their charts. Past medical history and smoking history were also obtained from the preoperative history and physical documentation. We defined current smokers as smokers who either still smoked or quit within three months of the surgical date. Former smokers include those patients who quit smoking for at least three months prior to surgery.

Primary outcomes for the study included postoperative complications, estimated blood loss (EBL), skin-to-skin procedure duration, chest tube duration, hospital LOS, and in-hospital mortality between SES groups. Mean and standard error of the mean (SEM), or else median and interquartile range (IQR), were used to report continuous variables. Number counts and percentages were used for categorical variables. Differences in means for continuous variables were compared using Student’s t-test or Wilcoxon rank-sum test (two groups), while differences in medians were compared using the Kruskal-Wallis test. We used the chi-square test or Fisher exact test to investigate the association between postoperative complications and other categorical variables.

Variables that significantly differed in univariate analyses were included in multivariable analyses. Logistic regression analysis with a stepwise selection procedure was used for the multivariable analysis of outcomes. Inverse Gaussian (V (μ) = μ3) regression model was utilized to evaluate predictive variables for chest tube duration and hospital LOS [17]. Because the inverse Gaussian model uses log-link function to transform the distribution to linear, the estimate of the parameter needs to be exponentiated for an explanation, and the value denotes the factor by which each variable increases the outcome.

Survival plots were generated using the Kaplan-Meier method, with survival between groups being compared using the log-rank statistic. Survival differences between the individual clinical and pathologic stages were determined using the Cox regression method.

Statistical analyses were performed using the SAS 9.4, 2013 (SAS Institute Inc., Cary, North Carolina (NC), USA). A p-value of ≤0.05 were considered to indicate statistical significance.

## Results

Demographics and preoperative comorbidities

Our study population comprised 437 patients, of which there were 186 (42.6%) men and 251 (57.4%) women. The mean age at surgery was 67.5 years, ranging from 24 to 87 years. There were 50 patients (11.4%) with an SES below 3x the poverty level (Group 1) and 387 (88.5%) patients with an SES above 3x the poverty level (Group 2). Preoperative pulmonary function tests showed that patients in Group 1 tended to have a lower FEV1% than patients in Group 2, but this difference did not reach significance (p=0.068), with FEV1% averaging 82.3% and 87.7%, respectively (Table 1).

**Table 1:** Patient Demographics SES: socio-economic status; 3x Poverty: three-times federal poverty level; SEM: standard error of mean; BMI: body mass index; BSA: body surface area; FEV1%: forced expiratory volume in one second as percent of predicted

Patient Characteristics		Total (n = 437)	SES Below 3x Poverty (n=50)			SES Above 3x Poverty (n=387)	p-value	
Age, yr; mean ± SEM	67.5 ± 0.47		66.4 ± 1.4			67.7 ± 0.5	0.380	
BMI, kg/m^2^; mean ± SEM	28.0 ± 0.3			28.2 ± 0.7	28.0 ± 0.3		0.773	
BSA, m^2^; mean ± SEM	1.89 ± 0.01			1.88 ± 0.03	1.89 ± 0.01		0.904	
FEV1%, mean ± SEM	87.1 ± 0.9			82.3 ± 2.8	87.7 ± 1.0		0.068	
Gender, n (%)	-			-	-		0.827	
Male	186 (42.6%)			22 (44.0%)	164 (42.4%)		-	
Female	251 (57.4%)			28 (56.0%)	223 (57.6%)		-	
Smoking Status	-			-	-		0.154	
Current smokers	140 (32.0%)			22 (44.0%)	118 (30.5%)		-	
Former smokers	218 (49.9%)			21 (42.0%)	197 (50.9%)		-	
Never	79 (18.1%)			7 (14.0%)	72 (18.6%)		-	

When comparing preoperative comorbidities and smoking status between the two SES groups, only preoperative coronary artery disease (CAD) or myocardial infarction (MI) (26.0% vs 15.0%, p=0.047) and preoperative COPD (32.0% vs 19.4%, p=0.039) were significantly different between patients with SES below 3x poverty and those with SES above 3x poverty (Tables 1, 2). There were no significant differences in tumor size (p=0.758), histology (p=0.690), or pathologic stage (p=0.802) between the two SES groups (Table 3).

**Table 2:** Smoking Status and Comorbidities * statistically significant (p≤0.05) 3x Poverty: three-times federal poverty level; COPD: chronic obstructive pulmonary disease; CAD: coronary artery disease; MI: myocardial infarction; CVA: cerebrovascular accident; GERD: gastroesophageal reflux disease

Patient Comorbidities	Total (n = 437)	Below 3x Poverty (n = 50)	Above 3x Poverty (n = 387)	p-value
COPD	91 (20.8%)	16 (32.0%)	75 (19.4%)	0.039*
Asthma	33 (7.5%)	1 (2.0%)	32 (8.3%)	0.155
Heart valvular disease/cardiomyopathy	29 (6.6%)	5 (10.0%)	24 (6.2%)	0.358
CAD or MI	71 (16.2%)	13 (26.0%)	58 (15.0%)	0.047*
CVA	18 (4.1%)	4 (8.0%)	14 (3.6%)	0.138
Carotid stenosis	22 (5.0%)	3 (6.0%)	19 (4.9%)	0.729
Congestive heart failure	8 (1.8%)	1 (2.0%)	7 (1.8%)	1.000
Obstructive sleep apnea	30 (6.9%)	2 (4.0%)	28 (7.2%)	0.558
Pulmonary embolism	19 (4.3%)	3 (6.0%)	16 (4.1%)	0.468
Prior pneumonia	39 (8.9%)	4 (8.0%)	35 (9.0%)	1.000
Pulmonary fibrosis	5 (1.1%)	0 (0.0%)	5 (1.3%)	1.000
Cirrhosis/liver failure	2 (0.4%)	1 (2.0%)	1 (0.3%)	0.216
Diabetes mellitus	76 (17.4%)	10 (20.0%)	66 (17.1%)	0.605
GERD	87 (19.9%)	9 (18.0%)	78 (20.2%)	0.719
Kidney disease	15 (3.4%)	2 (4.0%)	13 (3.4%)	0.685
Chronic anemia	11 (2.5%)	1 (2.0%)	10 (2.6%)	1.000
Coagulation disorders, hemophilias, thrombocytopenia	6 (1.4%)	0 (0.0%)	6 (1.6%)	1.000
Previous cancers	185 (42.3%)	21 (42.0%)	164 (42.4%)	0.960
Hypertension	248 (56.6%)	33 (66.0%)	215 (55.6%)	0.161
Hyperlipidemia	209 (47.8%)	22 (44.0%)	187 (48.3%)	0.565
Atrial fibrillation	29 (6.6%)	1 (2.0%)	28 (7.2%)	0.230
Other arrhythmias	20 (4.6%)	3 (6.0%)	17 (4.4%)	0.490
Peripheral vascular disease	17 (3.9%)	4 (8.0%)	13 (3.4%)	0.117
Pancreatitis	6 (1.4%)	1 (2.0%)	5 (1.3%)	0.520

**Table 3:** Tumor Characteristics ‡ Benign or lymphoma 3x Poverty: three-times federal poverty level; SEM: standard error of mean

Tumor Characteristics		Total (n = 437)		Below 3x Poverty (n = 50)		Above 3x Poverty (n = 387)	p-value
Tumor size, cm; mean ± SEM	3.3 ± 0.1		3.2 ± 0.2		3.3 ± 0.1		0.758
Pathology, n (%)							
Primary lung cancer	405 (92.7%)		46 (92.0%)		359 (92.8%)		0.690
Pulmonary metastasis	28 (6.4%)		4 (8.0%)		24 (6.2%)		-
Other pathology‡	4 (0.9%)		0 (0.0%)		4 (1.0%)		-
Pathologic stage for primary lung cancer, n (%)							
Stage IA	168 (38.4%)		16 (32.0%)		152 (39.3%)		0.802
Stage IB	53 (12.1%)		6 (12.0%)		47 (12.1%)		-
Stage IIA	59 (13.5%)		6 (12.0%)		53 (13.7%)		-
Stage IIB	28 (6.4%)		6 (12.0%)		22 (5.7%)		-
Stage IIIA	80 (18.3%)		10 (20.0%)		70 (18.1%)		-
Stage IIIB	6 (1.4%)		1 (2.0%)		5 (1.3%)		-
Stage IV	11 (2.5%)		1 (2.0%)		10 (2.6%)		-

The incidence of various intraoperative complications was comparable between the SES groups, with no significant differences (Table 4). The rate of overall intraoperative complications, such as bleeding, phrenic or recurrent laryngeal nerve injury, bronchial injury, or diaphragmatic injury, was 8% for Group 1 and 6% for Group 2 (p=0.373). Overall conversion rates to open lobectomy were also similar, with 4% of patients in Group 1 and 6% of patients in Group 2 requiring conversion to open lobectomy (p=0.755) (Table 4).

**Table 4:** Intraoperative Complications 3x Poverty: three-times federal poverty level

Complication Variable	Total (n = 437)	Below 3x Poverty (n = 50)	Above 3x Poverty (n = 387)	p-value
Overall Intraoperative Complications	27 (6.2%)	4 (8.0%)	23 (5.9%)	0.373
Pulmonary Artery (PA) Bleeding	12 (2.7%)	2 (4.0%)	10 (2.6%)	0.408
Pulmonary Vein (PV) Bleeding	5 (1.1%)	1 (2.0%)	4 (1.0%)	0.457
Recurrent Laryngeal Nerve Injury	3 (0.7%)	0 (0.0%)	3 (0.8%)	0.694
Other Bleeding	1 (0.2%)	0 (0.0%)	1 (0.3%)	0.886
Phrenic Nerve Injury	1 (0.2%)	0 (0.0%)	1 (0.3%)	0.886
Bronchial Injury	5 (1.1%)	1 (2.0%)	4 (1.0%)	0.457
Diaphragm Injury	1 (0.2%)	0 (0.0%)	1 (0.3%)	0.886

Skin-to-skin duration in minutes and EBL did not differ significantly between the two SES groups (Table 5). Group 1 did tend to have a higher rate of postoperative complications, with 27 (54%) of the 50 patients experiencing complications compared to 133 (34%) of the 387 patients in Group 2 (p=0.007) (Table 5). Among the postoperative complications, the lower SES group had greater numbers of total postoperative complications and of prolonged air leaks for ≥5 days (p=0.007 and 0.044, respectively) than the higher SES group (Table 6). Median chest tube duration was significantly longer in Group 1 than in Group 2 (five days vs. four days, respectively) (p=0.032) (Table 5). The same was true for median hospital LOS, which was five days in Group 1 versus four days in Group 2 (p=0.034). In-hospital mortality for Groups 1 and 2, which were 0% (0 of 50) and 1.6% (6 of 387), respectively, did not differ significantly (p=1.00) (Table 5).

**Table 5:** Primary Outcomes ‡ Median (IQR) * statistically significant (p≤0.05) 3x Poverty: three-times federal poverty level; LOS: length of stay; IQR: Interquartile range

Outcomes	Socio-Economic Status		
	Below 3x Poverty	Above 3x Poverty	p-value
Skin-to-Skin Duration, min‡	177 (150-235)	178 (146-226)	0.756
Estimated Blood Loss, mL‡	150 (100-300)	150 (100-300)	0.494
Post-Operative Complications, n (%)	27 (54.0%)	133 (34.4%)	0.007*
Chest tube duration, days‡	5 (3-8)	4 (2-6)	0.032*
Hospital LOS, days‡	5 (4-9)	4 (3-7)	0.034*
In-Hospital Mortality, n (%)	0 (0.0%)	6 (1.6%)	1.000

**Table 6:** Postoperative Complications Detailed ‡ any arrhythmia other than atrial fibrillation * statistically significant (p≤0.05) 3x Poverty: three-times federal poverty level; w/wo: with or without

Complication Variables	Total (n = 437)	Below 3x Poverty (n = 50)	Above 3x Poverty (n = 387)	p-value
Overall post-operative complications	160 (36.6%)	27 (54.0%)	133 (34.4%)	0.007*
Pulmonary-related complications				
Prolonged air leak for >5 days	92 (21.1%)	16 (32.0%)	76 (19.6%)	0.044*
Prolong air leak for >7 days w/wo subcutaneous emphysema	84 (19.2%)	15 (30.0%)	69 (17.8%)	0.040*
Pneumonia	27 (6.2%)	5 (10.0%)	22 (5.7%)	0.233
Chyle leak	18 (4.1%)	3 (6.0%)	15 (3.9%)	0.447
Mucous plug requiring intervention	17 (3.9%)	1 (2.0%)	16 (4.1%)	0.706
Respiratory failure	8 (1.8%)	1 (2.0%)	7 (1.8%)	1.000
Hypoxia	5 (1.1%)	2 (4.0%)	3 (0.8%)	0.102
Pneumothorax after chest tube removal requiring intervention	8 (1.8%)	0 (0.0%)	8 (2.1%)	0.605
Aspiration	6 (1.4%)	1 (2.0%)	5 (1.3%)	0.520
Pulmonary embolism	2 (0.5%)	0 (0.0%)	2 (0.5%)	1.000
Cardiovascular complications				
Atrial fibrillation	47 (10.8%)	9 (18.0%)	38 (9.8%)	0.079
Other arrhythmia‡ requiring intervention	6 (1.4%)	0 (0.0%)	6 (1.6%)	1.000
Shock/multi-organ system failure (MOSF)	5 (1.1%)	1 (2.0%)	4 (1.0%)	0.457
Cardiopulmonary arrest	3 (0.7%)	0 (0.0%)	3 (0.8%)	1.000
Myocardial infarction (MI)	2 (0.5%)	0 (0.0%)	2 (0.5%)	1.000
Cerebrovascular accident (CVA)	1 (0.2%)	0 (0.0%)	1 (2.0%)	0.114

Univariate and multivariable analysis

Among baseline characteristics of patients with postoperative complications versus patients without postoperative complications, SES, age, gender, intraoperative EBL, preoperative COPD, FEV1%, hypertension, gastroesophageal reflux disease (GERD), and other arrhythmias (arrhythmias other than atrial fibrillation) were significantly associated with postoperative complications. Preoperative chronic anemia was associated with postoperative complications at a borderline significant level (Table 7).

**Table 7:** Baseline Characteristics of Patients with or without Postoperative Complications ‡ any arrhythmias other than atrial fibrillation * statistically significant (p≤0.05) SEM: standard error of mean; IQR: inter-quartile range; EBL: estimated blood loss; FEV1%: forced expiratory volume in one second as percent of predicted

V ariables	Complications (n = 160)	No complications (n = 277)	p-value
Age, year; mean ± SEM	68.9 ± 0.7	66.7 ± 0.6	0.024*
Body Mass Index, kg/m^2^; mean ± SEM	28.2 ± 0.5	27.9 ± 0.4	0.698
Body Surface Area, m^2^; mean ± SEM	1.92 ± 0.02	1.87 ± 0.02	0.071
Male Gender, n (%)	86 (53.8)	100 (36.1)	<0.001*
Low Socio-Economic Status, n (%)	27 (16.9)	23 (8.3)	0.007*
EBL*, mL; median (IQR)	200 (100-355)	150 (100-250)	0.020*
Skin-to-Skin Operative Time, min; median (IQR)	193 (157-233)	172 (143-215)	0.061
FEV1%*, mean ± SEM	82.5 ± 1.6	89.8 ± 1.1	<0.001*
Smoking, n (%)	-	-	0.118
Current	53 (33.1)	87 (31.4)	-
Former	86 (53.8)	132 (47.7)	-
Never	21 (13.1)	58 (20.9)	-
Preoperative Comorbidities, n (%)			
Coronary Artery Disease or Myocardial Infarct	32 (20.0)	39 (14.1)	0.106
Chronic Obstructive Pulmonary Disease	47 (29.4)	44 (15.9)	<0.001*
Cerebrovascular Accident	9 (5.6)	9 (3.3)	0.229
Heart Valvular Disease/Cardiomyopathy	11 (6.9)	18 (6.5)	0.879
Atrial fibrillation	14 (8.8)	15 (5.4)	0.177
Other Arrhythmias‡	12 (7.5)	8 (2.9)	0.026*
Carotid Stenosis	8 (5.0)	14 (5.1)	0.980
Congestive Heart Failure	2 (1.3)	6 (2.2)	0.716
Hypertension	104 (65.0)	144 (52.0)	0.008*
Hyperlipidemia	83 (51.9)	126 (45.5)	0.198
Peripheral Vascular Disease	5 (3.1)	12 (4.3)	0.530
Obstructive Sleep Apnea	14 (8.8)	16 (5.8)	0.236
Asthma	13 (8.1)	20 (7.2)	0.730
Pneumonia	18 (11.3)	21 (7.6)	0.195
Pulmonary Fibrosis	1 (0.6)	4 (1.4)	0.657
Pulmonary Embolism	9 (5.6)	10 (3.6)	0.320
Cirrhosis or Liver Failure	1 (0.6)	1 (0.4)	1.000
Pancreatitis	4 (2.5)	2 (0.7)	0.198
Gastro-Esophageal Reflux Disease	42 (26.3)	45 (16.3)	0.012*
Kidney Disease	8 (5.0)	7 (2.5)	0.171
Chronic Anemia	1 (0.6)	10 (3.6)	0.062
Coagulation, Hemophilias, Thrombocytopenia	2 (1.3)	4 (1.4)	1.000
Diabetes Mellitus	33 (20.6)	43 (15.5)	0.175
Prior Cancer	68 (42.5)	117 (42.2)	0.958

**Figure 1:**
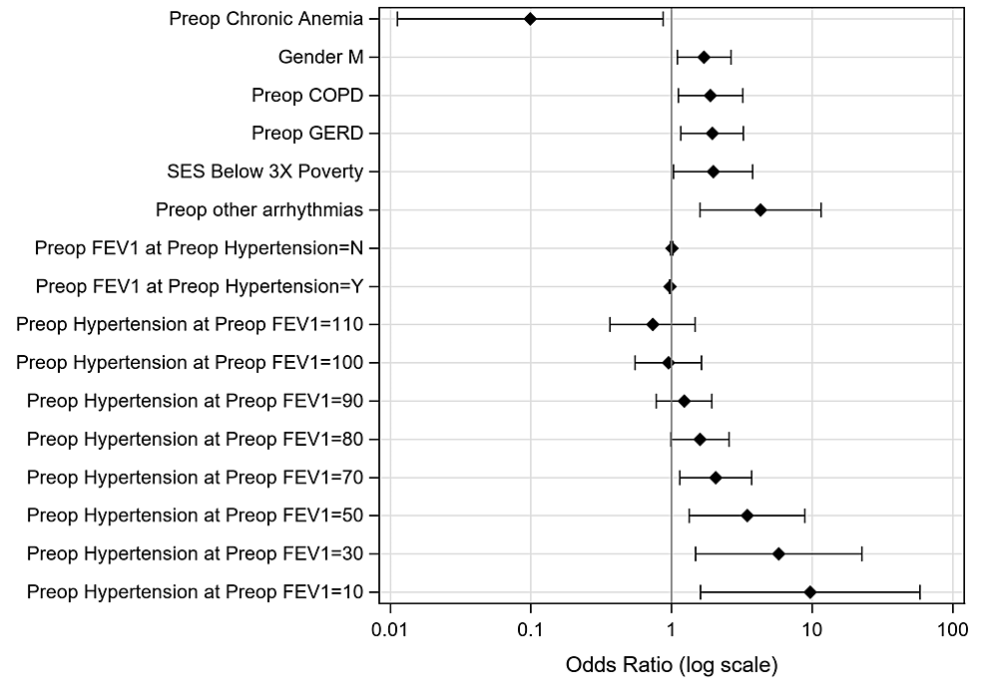
Plot of Odds Ratios for Predictors of Postoperative Complications Preop: preoperative; M: male; COPD: chronic obstructive pulmonary disease; GERD: gastro-esophageal reflux disease; SES: socio-economic status; 3x Poverty: three-times federal poverty level; FEV1: forced expiratory volume in one second

In multivariable logistical regression analysis (Table 8), the variables found to be independently positively associated with postoperative complications included Group 1 (below-3x-poverty) (Odds Ratio (OR)=1.98 vs. above-3x-poverty, 95%CI: 1.03-3.78), male gender (OR=1.70 vs female, CI: 1.10-2.64), preoperative COPD (OR=1.89, CI: 1.12-3.20), preoperative GERD (OR=1.94, CI: 1.16-3.24), preoperative other arrhythmias (OR=4.28, CI:1.59-11.55), and preoperative hypertension (OR=12.50, CI: 1.66-94.02). Thus, low SES (below 3x federal poverty) could increase the odds of postoperative complications independent of other covariates (Figure 1).

**Table 8:** Multi-Variable Logistic Regression Analysis on Predictors of Postoperative Complications ‡ any arrhythmia other than atrial fibrillation § Pre-Op FEV1%*Pre-Op Hypertension = interaction between preoperative FEV1% and preoperative hypertension CI: confidence interval; SES: socioeconomic status; 3x Poverty: three-times federal poverty level; EBL: estimated blood loss; FEV1%: forced expiratory volume in one second as percent of predicted; COPD: chronic obstructive pulmonary disease; GERD: gastroesophageal reflux disease

Variables	Odds Ratio (95% CI)	p-value
SES Below 3x Poverty	1.98 (1.03, 3.78)	0.039
Male Gender	1.70 (1.10, 2.64)	0.018
Age	1.02 (1.00, 1.05)	0.099
Estimated Blood Loss (EBL)	1.00 (1.000, 1.001)	0.092
Preoperative FEV1%	1.00 (0.99, 1.02)	0.775
Preoperative COPD	1.89 (1.12, 3.20)	0.017
Preoperative Hypertension	12.50 (1.66, 94.0)	0.014
Preoperative Other Arrhythmia‡	4.28 (1.59, 11.55)	0.004
Preoperative GERD	1.94 (1.16, 3.24)	0.011
Preoperative Chronic Anemia	0.10 (0.01, 0.87)	0.037
Preoperative FEV1%*Pre-Op Hypertension§	0.98 (0.96, 0.99)	0.026

Preoperative hypertension forms a negative interaction with preoperative FEV1%, suggesting that preoperative FEV1% would decrease the effect of pre-operative hypertension on the odds of postoperative complications. Preoperative chronic anemia was negatively associated with post-operative complications (OR=0.10, CI: 0.01-0.87).

Patients had significantly longer chest tube durations if they had lower SES (p=0.032), male sex (p=0.008), current and former smoking status (p=0.004), and preoperative COPD, CVA, pancreatitis, and GERD (Table 9). Chest tube duration negatively correlated with both preoperative FEV1% and BMI (r=-0.16, p=0.001; r=-0.10, p=0.045; respectively). In addition, patients with preoperative chronic anemia had significantly shorter chest tube durations (p=0.021) (Table 9).

**Table 9:** Differences in Chest Tube Duration and Hospital Length of Stay ‡ p-value: non-parametric Wilcoxon test * statistically significant (p≤0.05) IQR: interquartile range; LOS = length of stay; SES: socioeconomic status; 3x Poverty: three-times federal poverty level; COPD: chronic obstructive pulmonary disease; GERD: gastroesophageal reflux disease; CVA: cerebrovascular accident

Variables	Chest tube duration, days		LOS, days	
	Median (IQR)	p-value‡	Median (IQR)	p-value
SES	-	0.032	-	0.034*
Below 3x Poverty	5 (3-8)	-	5 (4-9)	-
Above 3x Poverty	4 (2-6)	-	4 (3-7)	-
Gender	-	0.008	-	0.006*
Male	4 (3-7)	-	5 (4-8)	-
Female	3 (2-6)	-	3 (2-6)	-
Smoking	-	0.004	-	0.001*
Current Smoker	4 (3-6.5)	-	5 (3-7.5)	-
Former Smoker	4 (3-6)	-	5 (3-8)	-
Never	3 (2-5)	-	4 (3-5)	-
Preoperative Comorbidities	-	-	-	-
COPD	5 (3-11)	<0.001	6 (4-10)	<0.001*
Hypertension	4 (3-7)	0.115	5 (3-8)	0.039*
Pancreatitis	14.5 (4-19)	0.048	9.5 (4-12)	0.048*
GERD	4 (3-8)	0.006	5 (3-8)	0.080
Chronic Anemia	2 (2-4)	0.021	4 (2-5)	0.055
Chemotherapy	5 (4-14)	0.073	6 (4-14)	0.029*
CVA	5.5 (4-9)	0.006	7 (5-9)	0.004*

Significantly longer hospital LOS was observed in patients of lower SES (p=.034), male sex (p=0.006), current and former smokers (p=0.001), and preoperative COPD, hypertension, CVA, pancreatitis, and chemotherapy (Table 10). Hospital LOS also negatively correlated with preoperative FEV1% and positively correlated with age (r=-0.17, p=0.0004 and r=0.10, p=0.033; respectively).

**Table 10:** Multi-Variable Inverse Gaussian Regression Model for Chest Tube Duration * statistically significant (p≤0.05) Exp(b): exponential function of parameter (b); CI: confidence interval; SES: socioeconomic status; 3x Poverty: three-times federal poverty level; BMI: body mass index; FEV1%: forced expiratory volume in one second as percent of predicted; COPD: chronic obstructive pulmonary disease; GERD: gastroesophageal reflux disease; CVA: cerebrovascular disease

Variables	Exp(b)	95% CI	p-value
Intercept	8.85	5.14-15.24	<0.001*
SES Below 3x Poverty	1.03	0.81-1.30	0.821
Male Gender	1.26	1.09-1.47	0.002*
BMI	0.98	0.97-1.00	0.006*
Preoperative FEV1%	1.00	0.99-1.00	0.067
Current Smoker	1.15	0.92-1.44	0.229
Former Smoker	1.03	0.86-1.23	0.784
Preoperative COPD	1.29	1.04-1.60	0.020*
Preoperative Pancreatitis	1.53	0.71-3.32	0.279
Preoperative GERD	1.34	1.10-1.62	0.003*
Preoperative Chronic Anemia	0.53	0.38-0.76	<0.001*
Preoperative CVA	1.29	0.85-1.96	0.227

**Figure 2:**
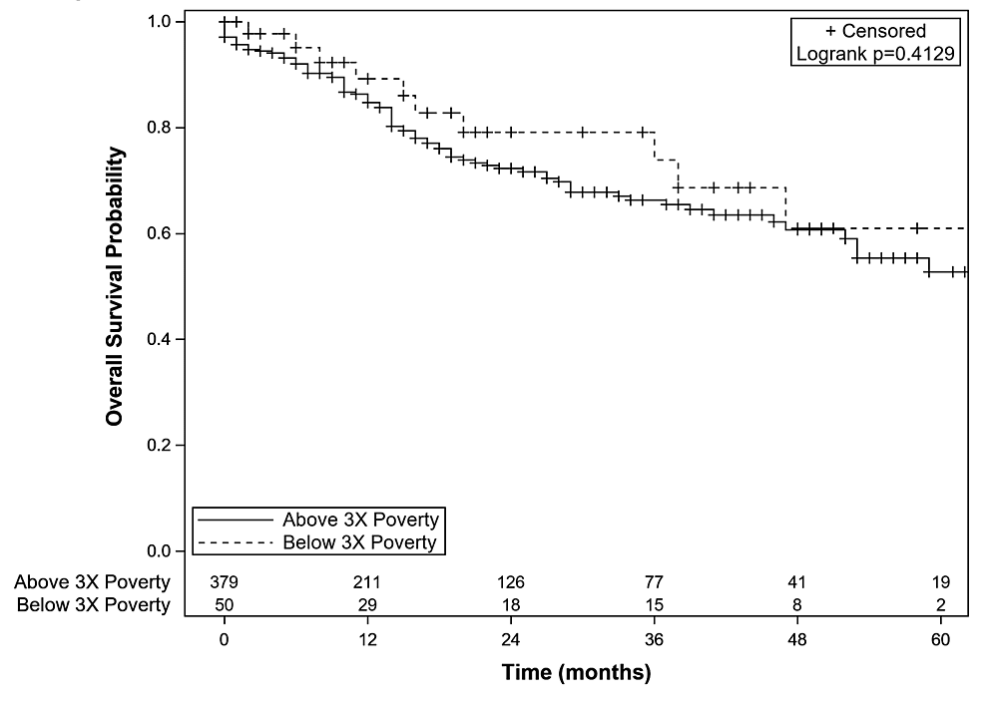
Kaplan-Meier Five-Year Overall Survival Curves Based on Socio-Economic Status 3x Poverty: three-times federal poverty level

Multi-variable analysis revealed that male gender, preoperative COPD, and preoperative GERD were independent predictors for longer chest tube duration, while BMI and preoperative chronic anemia were independent predictors for shorter chest tube duration (Table 11). Median hospital LOS for patients with preoperative COPD was 1.26 times greater than those without preoperative COPD (p<0.01). Significant positive predictors for hospital LOS also included age, preoperative FEV1%, current smoking status, and preoperative chemotherapy (Table 11). There was no significant difference in five-year OS between patients with SES below 3x poverty level and those with SES above 3x poverty level (p=0.413) (Figure 2).

**Table 11:** Multi-Variable Inverse Gaussian Regression Model for Hospital Length of Stay * statistically significant (p≤0.05) Exp(b): exponential function of parameter (b); CI: confidence interval; SES: socioeconomic status; 3x Poverty: three-times federal poverty level; Pre-Op: preoperative; FEV1%: forced expiratory volume in one second as percent of predicted; COPD: chronic obstructive pulmonary disease; CVA: cerebrovascular disease

Variables	Exp(b) †	95% CI†	p-value
Intercept	2.49	1.40-4.44	0.002*
SES† Below 3x Poverty†	1.16	0.94-1.43	0.174
male Gender	1.02	0.89-1.16	0.809
Age	1.01	1.01-1.02	<0.001*
Pre-Operative FEV1%†	1.00	0.99-1.00	0.047*
Current Smoker	1.28	1.06-1.56	0.012*
Former Smoker	1.02	0.88-1.18	0.804
Pre-Operative Hypertension	1.04	0.92-1.18	0.527
Pre-operative Chemotherapy	1.71	1.10-2.67	0.017*
Pre-operative COPD†	1.26	1.06-1.49	0.010*
Pre-operative Pancreatitis	1.24	0.67-2.31	0.490
Pre-Operative CVA†	1.15	0.80-1.63	0.453

## Discussion

The goal of this study was to investigate if and how SES influences outcomes for the population of patients undergoing MIS in the form of RAVT pulmonary lobectomy. As mentioned earlier, studies have shown that patients of lower SES are less likely to undergo MIS [6-9]. Patients of low SES have been found to be less willing to undergo minimally invasive procedures and are less likely to receive novel therapies in the setting of MI and minimally invasive revascularization procedures [18,19].

Findings from this study indicate that, when controlling for covariates, lower SES was predictive for a greater number of postoperative complications following RAVT pulmonary lobectomy. Lower SES has been found to have a significant influence on risk-adjusted mortality following pulmonary lobectomy, with the odds of death increasing with declining mean income [20]. This study highlights the importance of SES influence on outcomes following MIS for lung cancer.

Additionally, patients of low SES tend to have lower scores of self-reported health (SRH) [21]. Living in low neighborhood-level median income areas and having low educational attainment contribute to a significant risk in decline of SRH over time, even in patients who remain disease-free [21]. This implies that simply living in an impoverished area or having a low SES prompts patients to believe that they have worse health. These declines in SRH observed in patients with low SES were not nearly as prominent in patients of high SES and a greater level of educational attainment [21]. The significance of this association is that poor SRH has been identified as an independent predictor of increased LOS and mortality in patients undergoing cardiac surgery [22,23]. Thus, health perception can influence outcomes independently of other factors in a postsurgical setting and could have contributed to our findings in this study.

In a study conducted by Arpey, Gaglioti, and Rosenbaum (2017), the researchers conducted in-depth interviews with patients regarding their perception of how SES affects their healthcare. Most patients reported that their SES impacted the healthcare they receive. Some patients of low SES reported that physicians treated them differently based on their SES and that they felt ashamed and hesitant to return to care [24]. Similar to the findings associated with poor SRH, negative perceptions that patients of low SES can have about their own health, the treatment they receive, and their relationship with the provider could be contributing factors to worse outcomes even in the perioperative setting of minimally invasive procedures.

To address the outcomes found in our study, physicians performing RAVT could consider performing a SRH questionnaire for patients of low SES prior to surgery to identify those patients who might be at risk for increased perioperative complications. Considering that the population of patients of low SES who have access to and undergo MIS is small, paying special attention to this population with respect to cultivating the physician-patient relationship and gauging level of comfort and trust prior to surgery may prove beneficial with respect to outcomes.

Another potential contributing factor to the results of this study is the possibility that patients of low SES delayed their surgery further than patients of higher SES. The timeframe from diagnosis to surgery was not recorded in our study and could be a point of interest for future studies. This variable has significance, because lung cancer patients residing in ZIP codes of low median income have been shown to be more likely to delay surgical resection [25], and doing so has been associated with increased postoperative complications and increased perioperative mortality [26].

Our study also demonstrated preoperative COPD to be a positive predictive variable for postoperative complications, LOS, and chest tube duration following RAVT. Although there were no differences in smoking status between our patient groups, it is possible that the differences in preoperative COPD are explained by several factors associated with lower income. Second-hand smoke, occupational exposures (such as chemicals, fumes, vapors, and dusts), indoor air pollutants (such as biomass fuels and coal), outdoor air pollutants (prevalent in urban and high-income countries), and infections are all risk factors for developing COPD [27]. In addition to the aforementioned risk factors having an association with lower SES populations, poor populations often have a higher risk of developing COPD [28-30]. Additionally, the presence of preoperative COPD in lung cancer patients has been associated with worse outcomes following surgery, including a greater number of pulmonary complications and increased 30-day mortality [31].

An unexpected finding in our study was the significant predictive value of preoperative chronic anemia for decreased LOS and postoperative complications. Preoperative anemia has been associated with increased morbidity and mortality following major non-cardiac surgery [32] and decreased OS in NSCLC patients undergoing surgical resection [33]. It is possible that these patients received increased attention and management due to their anemia, which resulted in improved outcomes. These findings may be due to differences between those and our patient populations and warrant further study.

Limitations of this study also include the fact that it was retrospective. Our study was conducted at a specialized cancer center and, therefore, may not be generalizable to the public. The choice of using 300% of the federal poverty level for a single-family home may not have been entirely representative of the household sizes encountered in our study. Additionally, while there is evidence to support the use of ZIP code median income census data as a surrogate for SES, ZIP codes can contain SES heterogeneity within their populations [4,5,11,12].

## Conclusions

Lower ZIP-based median income as a surrogate for SES is associated with a greater number of postoperative complications following pulmonary resection for lung cancer after controlling for covariates. However, lower SES was not independently associated with greater chest tube duration, hospital LOS, in-hospital mortality, or OS. Thus, our study emphasizes the importance of SES awareness in the MIS setting and demonstrates that RAVT surgery is safe and feasible in patients of varying SES. Continued attention should be given to patients of low SES who undergo MIS, as they appear to be at increased risk for perioperative complications.
